# Porous Silica Gels Doped with Gold Nanoparticles: Preparation, Microstructure, Optical and Textural Properties

**DOI:** 10.3390/gels11060454

**Published:** 2025-06-13

**Authors:** Nina Danchova, Dimitar Shandurkov, Roumen Tsekov, Luben Mihaylov, Tony Spassov, Stoyan Gutzov

**Affiliations:** 1 Faculty of Chemistry and Pharmacy, Sofia University St. Kliment Ohridski, J. Bourchier Blvd. 1, 1164 Sofia, Bulgaria; ndanchova@chem.uni-sofia.bg (N.D.); fhds@chem.uni-sofia.bg (D.S.); tsekov@chem.uni-sofia.bg (R.T.); nhtlm@chem.uni-sofia.bg (L.M.); tspassov@chem.uni-sofia.bg (T.S.); 2 National Centre of Excellence Mechatronics and Clean Technologies, Sofia University St. Kliment Ohridski, 1164 Sofia, Bulgaria

**Keywords:** gold nanoparticles, silica, sol–gel, ceramic pigments, nanocomposites

## Abstract

Porous silica gel powders, doped with gold nanoparticles (AuNPs), were obtained by heating silica gels containing 1-dodecanethiol and tetrachloroauric acid at temperatures of 450 °C, 700 °C and 900 °C, and characterized using X-ray diffraction, TEM/EDS studies, UV/Vis reflectance spectroscopy and DTA/TG investigations. The color and microstructure of the obtained samples with a composition SiO_2_:AuNPs (about 0.03% Au) depend on the heating temperature. The UV/Vis reflection spectra of the samples are explained using Mie’s theory. The thermal stability of the obtained samples, as well as the processes occurring in the sol–gel matrix upon heating, were monitored by DTA/TG. The textural properties of the obtained materials were described based on adsorption–desorption isotherms. The obtained nanocomposites are promising pigments for ceramic glazes, similar to the Purple of Cassius. The textural properties of certain samples, **S_BET_** = 200–350 m^2^/g, a mean pore diameter (**D_AV_**) of approximately 10 nm and a specific pore volume (**V_t_**) between 0.5 and 0.8 cm^3^/g, make them promising candidates for catalytic applications, comparable to aerogel-like materials.

## 1. Introduction

Gold nanoparticles (AuNPs) exhibit unique optical properties due to their surface plasmon resonance (SPR), where conduction electrons on the particle surface collectively oscillate in response to specific light wavelengths. This phenomenon results in strong absorption and scattering of light, with the SPR peak’s position being influenced by factors such as particle size, shape, aggregation state and the surrounding medium’s refractive index. The incorporation of AuNPs into silica materials leads to the creation of nanocomposites, which can be applied as catalysts or ceramic pigments. One of the oldest and most prominent red glass pigments is Purple of Cassius, consisting of AuNPs in silicate matrices, using Sn^2+^ as a reduction agent [1,2,3]. The development of nanotechnology provides powerful instruments for the preparation of silica composites containing gold nanoparticles and using a wide variety of reduction agents or physicochemical techniques [4,5,6]. By combining nanotechnological approaches with Mie theory-based calculations, nanocomposites with tunable optical and textural properties can be obtained. Moreover, silica doped with AuNPs and SiO_2_:AuNPs is a promising multifunctional material with potential applications in photothermal therapy, drug delivery and cell bioimaging [7,8].

Sol–gel technology is a method for producing materials through a chemical process that involves the conversion of a solution (sol) into a solid (gel) phase. The process involves the hydrolysis and condensation reactions of metal alkoxides or metal salts in a liquid medium, usually water or alcohol. Sol–gel technology is used in a wide range of applications, including the production of ceramics, glasses, coatings, fibers and catalysts. One of its key advantages is the ability to form materials at relatively low temperatures, which is particularly beneficial for certain applications. An additional advantage of the sol–gel process lies in its ability to promote molecular-level homogenization of multicomponent systems, thereby enabling the formation of uniformly doped powders, as well as bulk xerogels or aerogels, depending on the selected drying method [9].

Recently, we developed a specific and efficient one-pot sol–gel method for preparing silica powders doped with gold nanoparticles (AuNPs), using 1-dodecanethiol, tetraethoxysilane and tetrachloroauric acid [4,7,8]. The reduction occurs upon heating and the resulting powders become irreversibly red–pink colored. In this way, a red–purple ceramic pigment is produced without the use of Sn^2+^ as a reducing agent, unlike in the traditional synthesis of Purple of Cassius [1,5]. In our previous studies, certain aspects, such as the porosity of the obtained powders and the temperature required for ceramic pigment formations, have remained open questions. From a physicochemical point of view, thermal treatment may induce crystallization of the silicate matrix, as well as agglomeration and coalescence of the gold nanoparticles, which could drastically alter the physical properties of the resulting nanocomposites. From the perspective of a physical description of the optical properties of ceramic pigments based on gold nanoparticles, the development of an appropriate algorithm—grounded in Mie theory for polydisperse powders—is more than necessary.

Many composites based on Au nanoparticles have been described in the literature. Pham et al. [10] studied the reduction of H[AuCl_4_] in epoxy resin. Studies show that the temperature plays a crucial role in the reduction and growth kinetics of the nanoparticles because an increase in the temperature can lead to an increase in the diffusion coefficients of the ions and particles [11,12].

Inorganic oxide composites containing Au nanoparticles is a field that does not cease its development. Many different matrixes have been reported, including SiO_2_, TiO_2_, Al_2_O_3_ [13,14] and more. Moreover, distinct types of composite materials have been produced, such as the following: nanoparticles encapsulated in hollow oxide particles [15,16], spheres functionalized with nanoparticles of their surface [17] and glasses and gels containing nanoparticles [18]. Many of these materials are used as a catalyst for different reactions. Singh et al. have tested such composites (Au@TiO_2_) as a catalyst for the photodegradation of methylene blue, malachite green and methyl orange [13]. Others have described that AuNP composites find use in the oxidation of CO. Furthermore, the matrix in which the particles are embedded can have an effect on the activity of the catalyst [19].

Oxide materials containing Au nanoparticles demonstrate good activity for different reactions in electrochemistry and organic chemistry. CeO_2_ decorated with AuNPs has good activity and stability after long work periods for an oxygen evolution reaction [20]. Furthermore, AuNPs and their composites have demonstrated good activity for many reactions in organic chemistry, including the following: their addition to multiple C-C bonds, oxidation, reduction, cyclization, C-C coupling reactions and more [21]. Another advantage the composite materials have over the AuNPs sols is their reusability. In many cases, the composite materials can be recycled by centrifuge or filtering and washing. They can maintain their catalytic activity for many cycles [22,23].

This study builds upon our previous work and aims to provide a comprehensive analysis of the influence of temperature on the optical and textural properties of nanocomposites synthesized via a novel heating method. This approach employs 1-dodecanethiol, tetraethoxysilane and tetrachloroauric acid as precursors, leading to the formation of highly porous SiO_2_:AuNPs ceramic pigments upon heating.

## 2. Results and Discussion

Gold-doped silica powders were synthesized via a sol–gel route, based on 1-dodecanethiol, tetraethoxysilane and tetrachloroauric acid. Initially, 5.6 mL of 12 mM ethanolic solution of tetrachloroauric acid (HAuCl_4_) was prepared and used as the gold precursor. To this solution, 1.5 mL of absolute ethanol was added, and the mixture was stirred for 5 min. Subsequently, 5 mL of tetraethyl orthosilicate (TEOS) was introduced and stirred for an additional 5 min. Controlled hydrolysis was initiated by adding 0.4 mL of distilled water, followed by stirring for 1 h at room temperature to promote gel formation. Next, 0.54 mL of 1-dodecanethiol (DDT) was added to the mixture to facilitate in situ reduction and stabilization of gold nanoparticles. Then, a small amount of catalyst (3 mL) was added to accelerate the condensation reactions within the sol–gel network. The chemical composition of the catalyst solution used here is H_2_O, C_2_H_5_OH and NaOH. It is a typical base gelation catalyst, used in sol–gel chemistry to accelerate the condensation process. As it has been pointed out in our recent contributions [2,3], the use of an ammonia base catalyst leads to the formation of Au-NH_3_ complexes; this is why NaOH was used here. On the other hand, the use of NaOH leads to the formation of NaCl traces in the final gel, visible in the X-ray diffraction diagrams of the heated and non-heated gels. The scheme used here led to reproducible transparent sol and gel products. Slow gelation results in the participation of gold microcrystals on the walls of the plastic reactor or on the bottom of the gel species. The pH of the final solution, after the gelation catalyst addition, was 7.5. The preparation of the samples was conducted under air. The samples were left to age for 48 h in sealed reactors, after which the drying was carried out in open reactors under ambient air at room temperature; then, bulk non-uniform gels were obtained.

**Figure 1:**
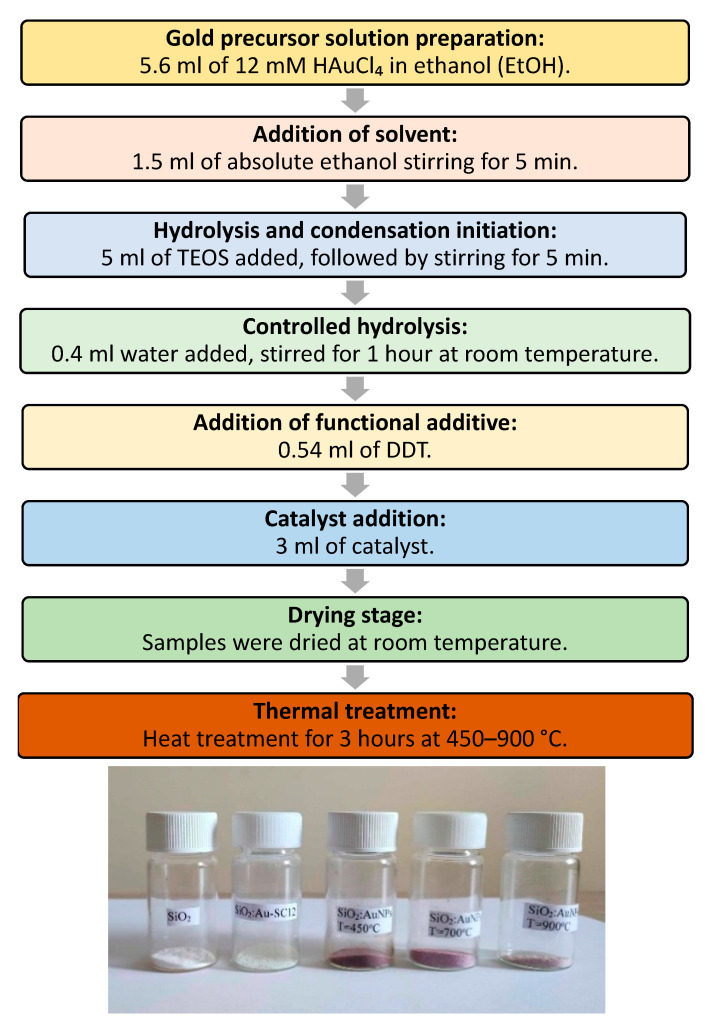
Preparation scheme for SiO_2_:AuNPs upon heating, starting with SiO_2_:AuSC_12_H_25_ and photographs of the obtained ceramic pigments.

Finally, a thermal treatment was performed by heating the pulverized samples at temperatures ranging from 450 °C to 900 °C for 3 h, leading to the formation of silica-based powders containing embedded gold nanoparticles. The schematic representation of the sol–gel synthesis route for AuNPs-doped silica materials is shown in Figure 1. In the described procedure, no gold loss on the vessel walls was observed.

It is evident that the color of the resulting composites depends on the preparative conditions. The composite containing the Au-SC_12_H_25_ complex (SiO_2_:Au-SC12) exhibits a yellowish hue, while the most intense red coloration is observed at a heating temperature of 450 °C. Powders obtained at temperature T = 900 °C display a weak pink coloration.

**Figure 2:**
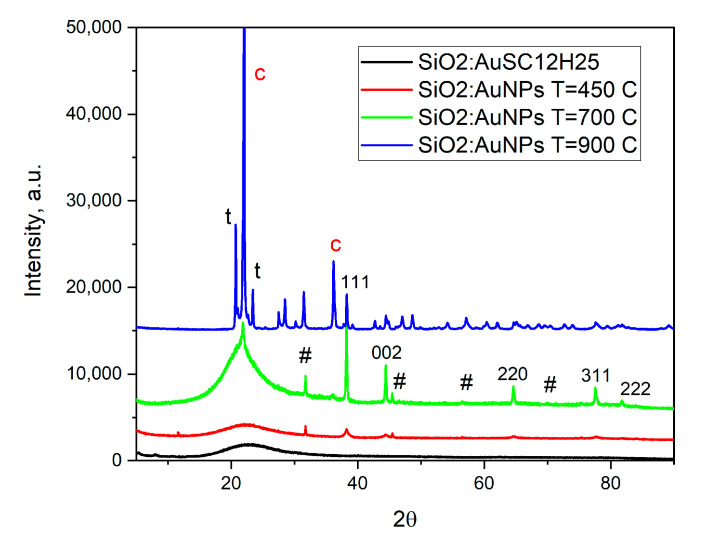
X-ray diffraction results of the investigated samples. Sample notations: NaCl 01–080–3939 traces are designated with #. Peaks from Au 01–073–9564 are indexed (hkl). The peaks from tetragonal low cristobalite (ICSD 34932) and monoclinic low tridymite (ICSD 176) are designated with **c** and **t**, respectively, (blue curve, T = 900 °C).

An X-ray diffraction study confirms that the SiO_2_:AuSC_12_H_25_ composites are amorphous at low temperatures (Figure 2). Upon heating to 450 °C, gold nanoparticles begin to form and grow with the increasing temperature, as indicated by the narrowing of diffraction peak widths. Traces of NaCl could also be detected. The mean particle sizes, calculated using the Scherrer equation for the (111) reflection of gold, are approximately 15 nm, 38 nm and 42 nm for samples heated at 450 °C, 700 °C or 900 °C, respectively. This increase in crystallite size with temperature is consistent with the known thermal behavior of gold nanoparticles. A phase analysis of the sample heated to 900 °C (Supplementary Materials) reveals the presence of 74% tetragonal low cristobalite (c, ICSD 34932) and 26% monoclinic low tridymite (t, ICSD 176) [24,25].

**Figure 3:**
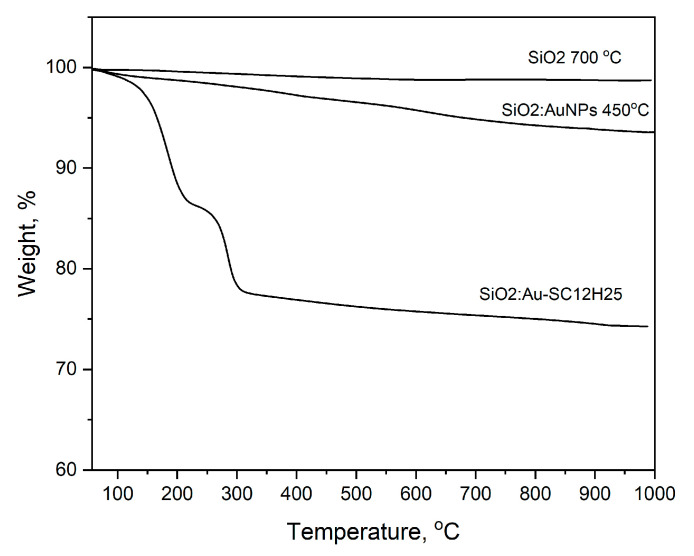
TG curves of a sample obtained by thermal treatment of 1-dodecanethiol embedded in a silicate gel (bottom curve). The upper curves represent the reference sample, consisting of pure silica, heated at 700 °C (GR0T), and of SiO_2_:AuNPs, obtained at 450 °C sintering.

The DTA/TG curves presented here (Figure 3) are consistent with our previous studies on silicate gels doped with gold nanoparticles [3,4,9]. Two distinct weight loss steps are observed, as follows: the first, occurring up to approximately 200 °C, is attributed to dehydration of the silicate matrix, which is a typical feature of sol–gel-derived materials; the second, around 300 °C, corresponds to the thermal decomposition of 1-dodecanethiol. These thermal profiles closely resemble those of silicate gels containing oleic acid and gold nanoparticles, as reported in our earlier work. However, the decomposition events in the present study are shifted to lower temperatures [3].

The thermogravimetric curve of the sample obtained at a heating temperature of 450 °C is very similar to that of pure silicon dioxide (SiO_2_) treated under the same conditions. The observed small weight losses (below 5%) are mainly due to residual organic compounds and adsorbed water retained within the porous structure of the material (330 m^2^/g).

**Figure 4:**
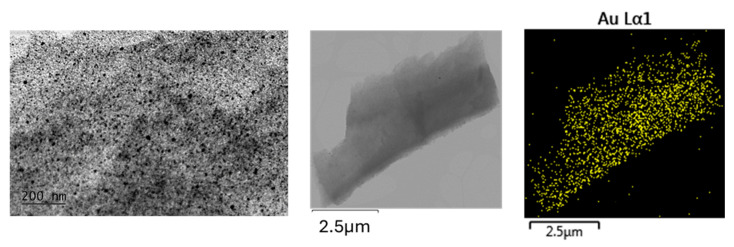
TEM image at 20k magnification and STEM—EDS of nanocomposites obtained at a temperature of T = 450 °C. The nanocrystallites exhibit sizes well below 40 nm.

**Figure 5:**
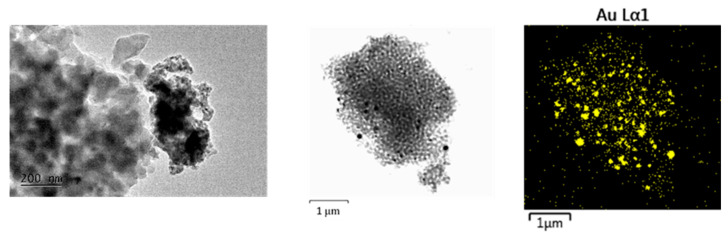
TEM at 20k magnification and STEM—EDS mapping microanalysis of gold nanoparticles dispersed in a silicate matrix. Heating temperature T = 700 °C.

**Figure 6:**
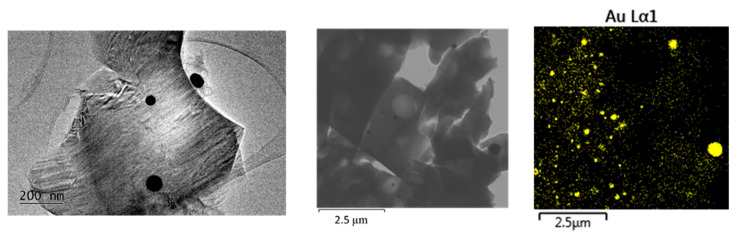
TEM at 20k magnification and STEM—EDS mapping microanalysis of different sized nanoparticles in crystalline SiO_2_:AuNPs, heating temperature T = 900 °C AuNPs.

The TEM results (Figure 4, Figure 5 and Figure 6) confirm the polydisperse nature of the synthesized optical material, both in terms of the optically active component and the gold nanoparticles (AuNPs) formed at elevated temperatures. Qualitatively, three distinct fractions of AuNPs with different sizes and morphologies are observed, dispersed within the amorphous silicate matrix, as follows:

- Fraction A—spherical particles below 40 nm (nanospheres);
- Fraction B—nanoparticles about 100 nm (nanospheres and nanoclusters);
- Fraction C—large, walled nanocrystals above 200 nm (giant nanocrystals).

At 900 °C, fractions B and C are dominating the nanoparticles distribution. However, even at this temperature, AuNPs smaller than 40 nm—and in some cases as small as 10 nm—are still observed. At 450 °C, fraction A is predominant, with numerous particles measuring less than 10 nm. At 700 °C, a mixture of all three fractions is present.

The formation of the nanocrystalline fraction C (giant nanocrystallites) can be explained by the melting and subsequent coalescence of gold nanoparticles, a process driven by the well-known phenomenon in nanoscience whereby smaller nanoparticles exhibit reduced melting temperatures.

**Figure 7:**
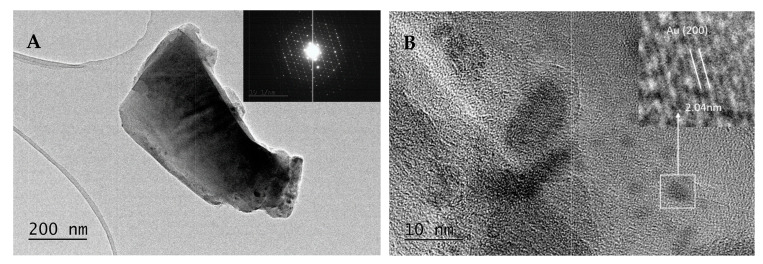
TEM micrograph (**A**) at 20k magnification and selected area electron diffraction (inset) and HRTEM (**B**) micrograph of crystalline SiO_2_:AuNPs, heating temperature T = 900 °C.

An electron diffraction study (SAED) clearly indicates the presence of monoclinic low trydimite in the cristobalite-rich regions (Figure 7). Large d-spaces are observed, at 4.8055 ($\bar{1}11$), 4.5511 (111) and 4.5896 ($\bar{4}02$), respectively. Such a finding is in accordance with the results in [26], suggesting a coexistence of crystobalite and trydimite fractions in crystallized silica. However, the results from HRTEM indicate that particles below 40 nm (fraction A) are still present.

**Figure 8:**
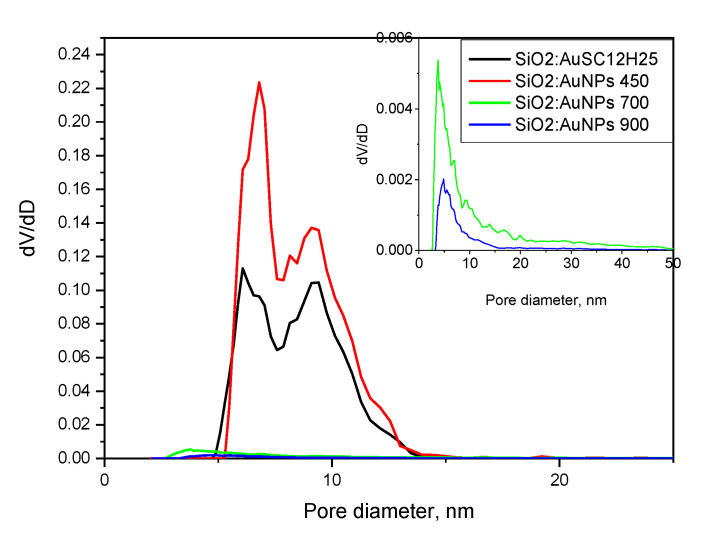
Pore size distribution curves for the samples. The heating temperature is shown. The samples heated at 700 and 900 °C are shown in the inlet for clarity.

**Figure 9:**
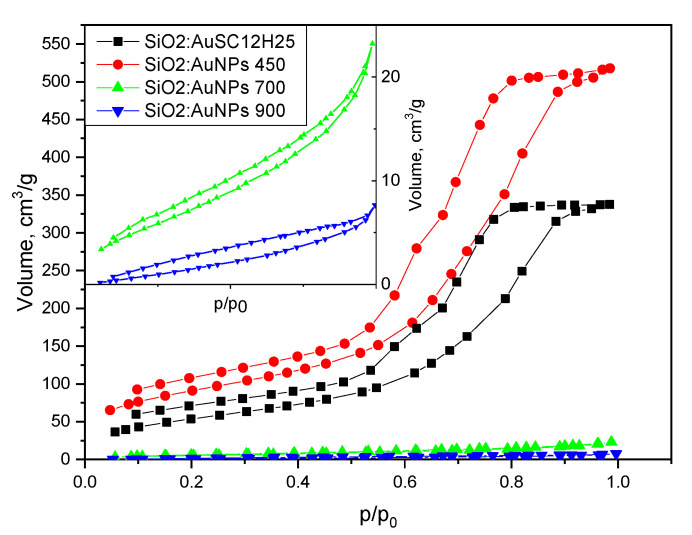
Textural properties of the investigated species. Samples heated at 700 and 900 °C have very low specific surface area and are shown in the inlet for clarity.

The textural properties of the heated gels, compared to those of the precursor (SiO_2_:AuSC_12_H_25_), are summarized in Table 1, Figure 8 and Figure 9. The initial material (SiO_2_:AuSC_12_H_25_) displays high mesoporosity, combined with a comparable specific surface area. The specific surface increases with heating at 450 °C because of thermal decomposition of the organic molecules. Sintering at temperatures of 750 °C and 900 °C leads to the densification of the powdered gels, together with a strong decrease in their porosity, specific surface and average pore diameter. The specific surface area is calculated using the BET isotherm.

The pore size distribution curves are shown in Figure 8. A bimodal distribution is observed for the non-heated sample and the sample heated at 450 °C.

**Figure 10:**
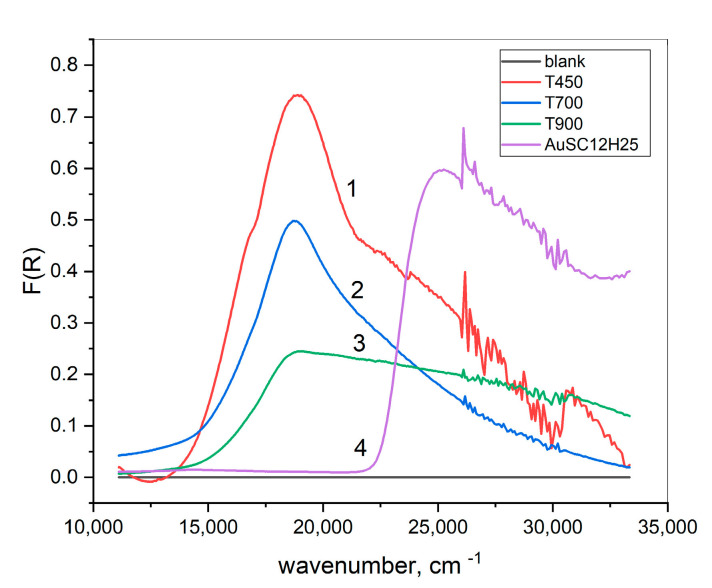
Differential reflectance spectra (reference SiO_2_ powder) of micropowders, heated at different temperatures. Samples: 1—SiO_2_:AuNPs, T = 450 °C; 2—SiO_2_:AuNPs, T = 450 °C; 3—T = 900 °C; 4—SiO_2_:AuSC12H25.

Figure 10 displays UV/Vis reflectance spectra of powders heated at different temperatures. In the spectra, a strong absorption peak at about 18 870 cm^−1^ (530 nm) is visible. This wavelength of 530 nm aligns with the typical range for the localized surface plasmon resonance (SPR) of spherical gold nanoparticles surrounded by silica.

It can be seen that the SPR peak at 530 nm consists of at least two Lorentzian components, which can be attributed to the size distribution of the AuNPs. This type of analysis can indeed offer deeper insights into the role of the silica matrix and the nanoparticle size dispersion in determining the optical properties [27]. The strong UV peak at about 25,000 cm^−1^ is assigned to electronic transitions in the yellow Au-SC_12_H_25_ complex.

Lowering the annealing temperature results in a pronounced increase in the intensity of the 530 nm peak. Assuming that the integral absorption intensity at 530 nm for the powders obtained at 450 °C is 100%, the corresponding intensities for the samples treated at 700 °C and 900 °C are approximately 60% and 25%, respectively.

However, the spectrum of nanocomposites obtained at a temperature of T = 900 °C differs significantly from the first two cases. The intensity of the 530 nm peak is notably reduced and accompanied by strong absorption in the ultraviolet region, comparable in magnitude to the 530 nm feature. This high-UV absorption for samples heated at 900 °C is likely due to defect states in the crystalline cristobalite-like matrix, as discussed in previous publications [3,28,29].

According to Mie’s theory, the red–violet color of AuNPs is attributed to the fraction with dimensions up to about 50 nm. The strength of the absorption peaks is influenced by the quantity of nanoparticles, whereas their color is determined by their size and the surrounding environment. Mie’s theory explains optical properties via the dielectric permittivity of AuNPs, which depends on the wavenumber x through the Drude model. In this case, the corresponding spectral intensity is well-approximated via the Lorentzian function [27,30].

$$h_{k}/[{1+4\left(x-x_{k}\right)}^{2}/{∆}_{k}^{2}]$$

where $h_{k}$ is the height of the *k*-th peak with a maximum at $x_{k}$ and a full-width at half-maximum (FWHM) ${∆}_{k}$. From the latter, one can calculate the mean free path $l_{k}=v_{F}/c{∆}_{k}$ of electrons in a gold nanoparticle, where c is the speed of light and $v_{F}=0.0046c$ is the Fermi velocity of gold. The maximum of the peak depends strongly on the medium around the particle via its dielectric constant $\varepsilon_{k}$

$$x_{k}^{2}=x_{p}^{2}/(\varepsilon_{k}+1)(2\varepsilon_{k}+1)-{∆}_{k}^{2}$$

where $x_{p}=72,000\;{cm}^{-1}$ is the Langmuir plasmon frequency of gold. Because $x_{k}$ is very high, only electrons can follow the resonant frequency in the surroundings. Thus, the corresponding high frequency dielectric constant $\varepsilon_{k}=n_{k}^{2}$ is equal to the square of the refractive index. Hence, one can calculate $n_{k}$ via Mie’s theory, as well from the experimental spectra [27].

**Figure 11:**
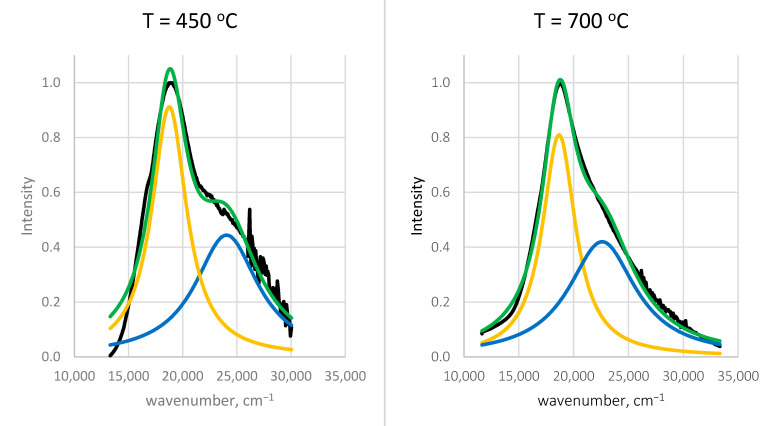
Deconvolution spectral results of normalized diffuse reflectance spectra for samples annealed at a different temperature. Black lines represent experimental data.

The spectra from Figure 11 are fitted using a superposition of two Lorentzian functions, each representing a population of gold nanoparticles with distinct sizes and surrounding environments.

The results of the conducted procedure are summarized in Table 2, as follows:

As shown in the figure, the two Lorentzian peaks are slightly affected by temperature. The first one corresponds to gold nanoparticles with a mean free path $l_{1}=12$ nm surrounded by a medium with a refractive index of $n_{1}=1.39$, while the second lower peak corresponds to $l_{2}=6$ nm and $n_{2}=1.16$. Obviously, the larger AuNPs are in close contact with the SiO_2_ matrix, which possesses a refractive index around n=1.46, while the smaller ones are distributed in some pores. The areas of the two Lorentzian peaks are commensurable, which means there is an equal volume fraction of the two kinds of gold nanoparticles.

Upon the heating of SiO_2_:Au-SC_12_H_25_, an irreversible decomposition reaction of Au-SC_12_H_25_ takes place, which produces AuNPs (Au°) and volatile organosulfur products. It follows that the sintering temperature plays a crucial role for the physical properties of the obtained nanocomposite. It is well-established that the formation of gold particles during reduction follows the Finke–Watzky mechanistic model, which is characterized by a slow nucleation, followed by a rapid chemical reaction and crystal growth [31,32]. In our case, the effect of temperature is two-fold, as follows: its’ increase induces the size-dependent melting of gold nanoparticles, followed by coalescence. More information about the size-dependent melting of nanocrystals can be found in [33].

As a result, there is a fraction of “giant nanocrystals” observable by TEM which do not contribute significantly to the absorption intensity of the SPR peak at 530 nm. Our results (Figure 4, Figure 5 and Figure 6) indicate that low heating temperatures prevent nanoparticle agglomeration, thereby increasing their concentration and consequently enhancing the absorption intensity in such samples. DDT and related products after oxidation by metal ions could adsorb on a fresh AuNPs’ surface and affect the dispersion properties as well [34].

The increase in sintering temperature also leads to a change in the silicate matrix. While at 450 °C the matrix is amorphous, at 900 °C it becomes crystalline (Figure 1, Table 1). In our view, this sample heated at 900 °C represents the first successful attempt to obtain cristobalite doped with gold nanoparticles. The occurrence of monoclinic low tridimite nanocrystals, accompanying the crystallization of cristobalite, is in accordance with the results published in [35,36,37]. From a general physicochemical point of view, the formation of metastable silica phases at cooling is explained by the Ostwald’s rule of stages [36]. The densification of the powders (Table 1, calculated densities) when they are heated is combined with a drastic decrease in their specific surface area and porosity, combined with a low-temperature crystallization of the silica matrix. The pore diameter decreases twice upon increasing the heating temperature, showing the matrix densification after heating. The amorphous samples here possess a high specific surface area (200–330 m^2^/g), typical of aerogel-like silicate materials, and show promise as potential catalysts or glaze pigments [38]. The catalytic properties of SiO_2_:AuNPS for different chemical reactions are well-known [38,39,40,41,42,43,44]. An intriguing trend is observed in Table 1 regarding the thermal behavior of the samples; upon heating to 450 °C, the specific surface area increases, likely due to the combustion of the organic component. A similar approach—thermal decomposition or evaporation of organic components—is commonly employed in the synthesis of mesoporous, thermally insulating aerogel materials, often utilizing natural raw materials [39]. At higher temperatures, however, a significant decrease in surface area occurs as a result of powder densification. This densification also affects the pore structure, with the average pore diameter decreasing from approximately 10 nm to around 5 nm. Furthermore, the pore size distribution curve shifts, indicating changes in the uniformity and range of pore sizes as a function of temperature.

**Figure 12:**
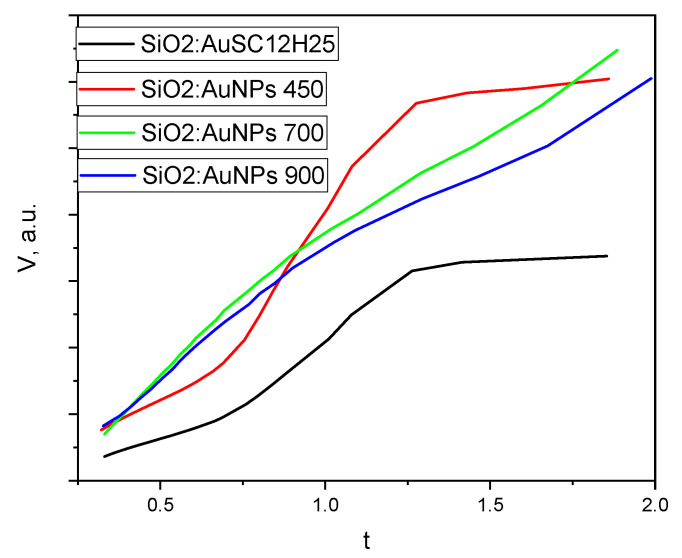
T-plot for the samples. Heating temperatures are shown.

An analysis of the nitrogen adsorption–desorption isotherms (Figure 8 and Figure 9) reveals that all samples exhibit hysteresis loops characteristic of Type IV isotherms, which are typical for mesoporous materials with pore diameters in the range of 2–50 nm (IUPAC classification) [45,46,47,48]. A t-plot was constructed using the de Boer standard isotherm (Harkins–Jura or de Boer model), as shown in Figure 12 [49,50]. The sample isotherm is plotted versus the standard isotherm and the deviations are interpreted in the following paragraphs. Both the non-heated sample and the sample annealed at 450 °C display H2(b)-type hysteresis loops [46], typically associated with complex pore structures and pore size polydispersity. This type of loop indicates a closed-pore morphology, often with “ink-bottle”-shaped pores, where narrow necks restrict desorption. Such structures result in complete pore filling at high relative pressures and steep desorption branches due to delayed evaporation from the pore interiors. These features are consistent with the pore size distribution curves (Figure 8), which show that the non-heated sample has an average pore diameter of 10 nm and a bimodal distribution with peaks at 6 and 9.4 nm. The sample annealed at 450 °C exhibits a slightly smaller average pore diameter (9.7 nm), with peaks centered at 6.8 and 9 nm. The reduced pore size contributes to the increased specific surface area observed in the thermally treated sample. In contrast, the samples annealed at 700 °C and 900 °C still exhibit Type IV isotherms, but with markedly different hysteresis loop types. These samples show significantly reduced specific surface areas (21 and 9 m^2^/g, respectively) and smaller average pore diameters (6.9 and 5 nm, respectively) compared to those treated at lower temperatures. The sample heated at 700 °C displays an H3-type hysteresis loop, commonly attributed to slit-like pores formed between plate-like particles or aggregates. The sample annealed at 900 °C exhibits an H4-type hysteresis, which shares similar structural origins with H3, but is generally associated with narrow slit-shaped pores and mesoporous materials. Notably, both samples lack saturation at high relative pressures, suggesting the presence of non-rigid porous frameworks that are capable of structural swelling [46]. The low closure points of the hysteresis loops further imply a broad pore size distribution. These interpretations are supported by the calculated t-curves (Figure 12).

The t-plots for SiO_2_:AuNPs and SiO_2_:AuNPs_450 show significant deviations from the linear trend at high relative pressures, consistent with mesoporous oxide materials exhibiting capillary condensation. In contrast, the t-curves for SiO_2_:AuNPs_700 and SiO_2_:AuNPs_900 conform relatively well to a straight line, with only minor deviations at intermediate and high pressures. The lower adsorbed volume at a high relative pressure might be connected to the presence of some mesopores in the gels. This near-linear behavior resembles that of a Type II isotherm, typically observed for non-porous or macroporous materials [46], and suggests metastability in the adsorbed multilayer and delayed condensation due to high pore curvature radii and a non-rigid gel structure. The findings are consistent with the phase analysis of the sample SiO_2_:AuNPs_900. Cristobalite and tridymite have layered structures and low specific surface areas due to microporosity—2 m^2^/g and 2,8 to 7.4 m^2^/g, respectively—together with a density of about 2.30 g/cm^3^ [51,52].

## 3. Conclusions

The results of this study underscore the critical impact of thermal treatment conditions on the nanoparticle size distribution, as well as the resulting optical and thermal properties of SiO_2_:AuNPs nanocomposites. The sol–gel synthesis method revealed herein, based on the co-doping of silica gels with 1-dodecanethiol and tetrachloroauric acid followed by thermal treatment, represents a promising route for the fabrication of glaze pigments or catalytic powders, depending on the specific processing parameters. Thermal treatment at a relatively low temperature (T = 450 °C) yields SiO_2_:AuNPs nanopowders characterized by a high specific surface area (330 m^2^/g) and an average pore diameter of 10 nm, closely resembling the textural features of silicate aerogels. This low-temperature approach offers a novel, cost-effective strategy for producing Au-doped silica powders with aerogel-like characteristics. Conversely, samples annealed at elevated temperatures (T = 900 °C) show the formation of crystalline phases, including tetragonal low cristobalite and monoclinic low tridymite, within which gold nanoparticles are embedded. Furthermore, the analysis of the optical spectra of these polydisperse powders demonstrates that employing Mie’s theory is a viable method for characterizing the optical behavior of such complex nanocomposite systems.

## 4. Materials and Methods

The following chemicals were used for the syntheses of the nanocomposite materials: absolute ethanol (EtOH), tetraethyl orthosilicate (TEOS), tetrachloroauric acid tri-hydrate (HAuCl_4_⋅3H_2_O), 1-dodecanethiol (DDT), sodium hydroxide (NaOH) and distilled water. All chemicals were supplied by Sigma-Aldrich (Darmstadt, Germany). The chemicals were of analytical grade. The catalyst employed in this study is composed of water (H_2_O), ethanol (EtOH) and sodium hydroxide (NaOH). Reflectance measurements were carried out using a PE Lambda 35 spectrometer (PerkinElmer LLC, 940 Winter St Waltham, MA, USA) equipped with a Spectralon^®^-coated integrating sphere. For all samples, the Kubelka–Munk function F(R) was calculated based on the diffuse reflectance R [53]. The spectral features were analyzed using Lorentzian fitting to determine the peak position (xc), full-width at half-maximum (FWHM) and integrated absorption intensity (A). A Labsphere Spectralon™ standard was used for reflectance calibration Diffuse Reflectance white and black standards SRS-99–010, SRS-02–010 [54] and a Ho_2_O_3_ powder were used as a reference.

The structure and microstructure of all samples were characterized by X-ray diffraction with Cu-Kα (1.5418 Å) radiation (Empyrean, Malvern Panalytical X-ray diffractometer), at a step of 2θ = 0.05° and counting time of 4 s/step. The mean crystallite sizes are calculated using Scherrer’s equation from the 111 peak of Au. The X-ray diffraction data are analyzed using the PowderCell software 2.3 [55].

TEM analyses were carried out using a JEOL JEM-2100 electron microscope operated at 200 kV with a LaB_6_ filament, equipped with a Gatan Orius 1000 CCD camera (JEOL, Akishima, Japan). STEM–EDS measurements were performed using an Oxford Instruments X-Max 80T detector (Oxford Instruments, Abingdon, UK). TEM image analysis was conducted using the ImageJ software (version IJ1.46r for 64bit Windows OS and Java 8) [56]. Over 20 images containing a total of more than 300 crystals were analyzed.

The textural properties of the aerogel powders were determined by low-temperature (77.4 K) nitrogen adsorption using a NOVA 1200e surface area and porosity analyzer (Quantachrome Instruments, Boynton Beach, FL, USA). Nitrogen adsorption–desorption isotherms were analyzed to determine specific surface areas (S_BET_) using the BET method, while total pore volumes (V_t_) and average pore diameters (D_v_) were estimated at a relative pressure of approximately 0.99. Prior to measurements, all samples were degassed under vacuum at 150 °C for 16 h. The pore size distribution (PSD) curves were calculated using Nonlocal Density Functional Theory (NLDFT) [57].

## Figures and Tables

**Table 1:** Textural properties of the investigated gels. The doping level of Au is 0.03% in all samples. The densities ρ_calc_ are obtained using the values for amorphous silica and cristobalite, 2.19 g/cm^3^ and 2.30 g/cm^3^, respectively.

Sample	S_BET_, m^2^/g	Vt, cm^3^/g	Dav nm	T °C	ρ_calc_ g/cm^3^
SiO_2_:AuSC_12_H_25_	204	0.52	10.0	-	1.02
SiO_2_:AuNPs	330	0.80	9.7	450	0.8
SiO_2_:AuNPs	21	0.04	6.9	700	2.01
SiO_2_:AuNPs	9	0.01	5.0	900	2.24

**Table 2:** Photophysical parameters of the investigated nanocomposites extracted from their diffuse reflectance spectra.

T [°C]	$h_{1}$	$x_{1}$ [cm^−1^]	${∆}_{1}$ [cm^−1^]	$h_{2}$	$x_{2}$ [cm^−1^]	${∆}_{2}$ [cm^−1^]
450	0.91	18,761	3911	0.44	24,048	7095
700	0.81	18,668	3666	0.42	22,599	7457

## Data Availability

The original contributions presented in this study are included in the article/Supplementary Materials. Further inquiries can be directed to the corresponding author.

## Supplementary Materials

The following supporting information can be downloaded at: https://www.mdpi.com/article/10.3390/gels11060454/s1.

## References

[1] Habashi F. (2016). Purple of Cassius: Nano Gold or Colloidal Gold?. Eur. Chem. Bull..

[2] Shandurkov D., Danchova N., Spassov T., Petrov V., Tsekov R., Gutzov S. (2023). Silica gels doped with gold nanoparticles: Preparation, structure and optical properties. Gels.

[3] Danchova N., Tsekov R., Shandurkov D., Gutzov S., Lyubenova L., Mihaylov L., Spassov T. (2025). Silica gels doped with gold nanoparticles and gold thiolate complexes: The effect of heating and preparation conditions. J. Non. Cryst. Solids.

[4] Ong C., Cha B.G., Kim J. (2019). Mesoporous Silica Nanoparticles Doped with Gold Nanoparticles for Combined Cancer Immunotherapy and Photothermal Therapy. ACS Appl. Bio Mater..

[5] Brust M., Walker M., Bethell D., Schiffrin D.J., Whyman R. (1994). Synthesis of thiol derivatised gold nanoparticles in a two phase liquid liquid system. Chem. Soc. Chem. Commun..

[6] Matsuoka J., Mizutani R., Nasu H., Kamiya K. (1992). Preparation of Au-Doped Silica Glass by Sol-Gel Method. J. Ceram. Soc. Jpn..

[7] Georgiev P., Chanachev A., Simeonova S., Ivanova T., Balashev K. (2020). A new method for studying the kinetics of synthesis of gold nanoparticles in hexadecylanilin monolayer at the air/water interface by means of atomic force microscopy. Comptes Rendus L’Academie Bulg. Sci..

[8] Georgiev P., Simeonova S., Tsekov R., Balashev K. (2020). Dependence of Plasmon Spectra of Small Gold Nanoparticles from Their Size: An Atomic Force Microscopy Experimental Approach. Plasmonics.

[9] Brinker C.J., Scherer G.W. (1990). Sol-Gel Science: The Physics and Chemistry of Sol-Gel Processing.

[10] Pham Q.T., Ngo G.L., Nguyen X.A., Nguyen C.T., Ledoux-Rak I., Lai N.D. (2023). Direct Synthesis of Gold Nanoparticles in Polymer Matrix. Polymers.

[11] Ozkaraoglu E., Tunc I., Suzer S. (2009). Preparation of Au and Au–Pt nanoparticles within PMMA matrix using UV and X-ray irradiation. Polymer.

[12] Kudryashov A., Baryshnikova S., Gusev S., Tatarskiy D., Lukichev I., Agareva N., Poddel’sky A., Bityurin N. (2022). UV-Induced Gold Nanoparticle Growth in Polystyrene Matrix with Soluble Precursor. Photonics.

[13] Singh J., Soni R.K. (2021). Tunable optical properties of Au nanoparticles encapsulated TiO_2_ spheres and their improved sunlight mediated photocatalytic activity. Colloids Surf. A Physicochem. Eng. Asp..

[14] Díaz C., Cifuentes-Vaca O., Valenzuela M.L. (2023). Matrix Effect of Properties of Au, ZnO and Eu_2_O_3_: Silica, Titania and Alumina Matrices. Micro.

[15] Cavaliere-Jaricot S., Darbandi M., Nann T. (2007). Au–silica nanoparticles by “reverse” synthesis of cores in hollow silica shells. Chem. Commun..

[16] Lu X., Mei T., Guo Q., Zhou W., Li X., Chen J., Zhou X., Sun N., Fang Z. (2018). Improved performance of lateral flow immunoassays for alpha-fetoprotein and vanillin by using silica shell-stabilized gold nanoparticles. Microchim. Acta.

[17] Kim J., Park C., Kim Y. (2022). Hollow Au nanoparticles-decorated silica as near infrared-activated heat generating nano pigment. J. Ind. Eng. Chem..

[18] Zanella R., Sandoval A., Santiago P., Basiuk V.A., Saniger J.M. (2006). New Preparation Method of Gold Nanoparticles on SiO_2_. J. Phys. Chem. B.

[19] Ishida T., Murayama T., Taketoshi A., Haruta M. (2020). Importance of Size and Contact Structure of Gold Nanoparticles for the Genesis of Unique Catalytic Processes. Chem. Rev..

[20] Park S., Nguyen Q.T., Choi J., Park J.H., Park J.R., Nakate U.T., Park S. (2023). Au-nanoparticles-decorated CeO_2_ electrocatalyst synthesized by direct growth and wet impregnation for enhanced oxygen evolution reaction. Surf. Interfaces.

[21] Corma A., Garcia H. (2008). Supported gold nanoparticles as catalysts for organic reactions. Chem. Soc. Rev..

[22] Murtaza A., Uroos M., Sultan M., Muazzam R., Naz S. (2021). Enhancing catalytic potential of gold nanoparticles by linear and cross-linked polyurethane blending. RSC Adv..

[23] Padikkaparambil S., Narayanan B., Yaakob Z., Viswanathan S., Tasirin S.M. (2013). Au/TiO_2_ Reusable Photocatalysts for Dye Degradation. Int. J. Photoenergy.

[24] Peacor D.R. (1973). High-temperature single-crystal study of the cristobalite inversion. Z. Fuer Krist..

[25] Kato K., Nukui A. (1976). Die Kristallstruktur des monoklinen Tief-Tridymits. Acta Crystallogr. Sect. B.

[26] Konon M., Polyakova I.G., Mazur A.S., Saratovskii A.S., Danilovich D.P., Alikin M. (2023). Crystallization of Cristobalite in Sodium Borosilicate Glass in the Presence of Cr_2_O_3_. Materials.

[27] Kreibig U., Vollmer M. (1995). Optical Properties of Metal Clusters.

[28] Gutzov S., Bredol M. (2006). Optical properties of cerium and terbium doped silica xerogels. J. Mat. Sci..

[29] Nesheva D., Levi Z., Aneva Z., Nikolova V., Hofmeister H. (2000). Experimental studies on the defect states at the interface between nanocrystalline CdSe and amorphous SiO_x_. J. Phys. Condens. Matter.

[30] Fan X., Zheng W., Singh D.J. (2014). Light scattering and surface plasmons on small spherical particles. Light Sci. Appl..

[31] Bentea L., Watzky M.A., Finke R.G. (2017). Sigmoidal Nucleation and Growth Curves Across Nature Fit by the Finke–Watzky Model of Slow Continuous Nucleation and Autocatalytic Growth: Explicit Formulas for the Lag and Growth Times Plus Other Key Insights. J. Phys. Chem. C.

[32] Watzky M.A., Finke R.G. (1997). Transition Metal Nanocluster Formation Kinetic and Mechanistic Studies. A New Mechanism When Hydrogen Is the Reductant: Slow, Continuous Nucleation and Fast Autocatalytic Surface Growth. J. Am. Chem. Soc..

[33] Buffat P., Borel J.-P. (1976). Size effect on the melting temperature of gold particles. Phys. Rev. A.

[34] Chegel V., Rachkov O., Lopatynskyi A., Ishihara S., Yanchuk I., Nemoto Y., Hill J.P., Ariga K. (2012). Gold Nanoparticles Aggregation: Drastic Effect of Cooperative Functionalities in a Single Molecular Conjugate. J. Phys. Chem. C.

[35] Gutzow I., Pascova R., Jordanov N., Gutzov S., Petkov I., Markovska I., Schmelzer J.W.P., Ludwig F.P., Schmelzer J.W.P. (2014). Crystalline and Amorphous Modifications of Silica: Structure, Thermodynamic Properties, Solubility and Synthesis in “Glass”.

[36] Gutzow I.S., Schmelzer J.W.P. (1994). The Vitreous State.

[37] Perrotta A.J., Grubbs D.K., Martin E.S., Dando N.R., McKinstry H.A., Huarg C.Y. (1989). Chemical Stabilization of β-Cristobalite. J. Am. Ceram. Soc..

[38] Coppage R., Leopold M., Allen G., Lacy C. (2017). Gold Nanoparticle in Ceramic Glaze 2018. U.S. Patent.

[39] Deng Y., Sha Z., Wang X., Duan K., Xue W., Beadham I., Xiao X., Zhang C. (2025). Exploration of Key Factors in the Preparation of Highly Hydrophobic Silica Aerogel from Rice Husk AshAssisted by Machine Learning. Gels.

[40] Csupász-Szabó H.J., Döncző B., Szarka M., Daróczi L., Lázár I. (2025). Thermal Reverse-Engineered Synthesis and Catalytic Activity of Nanogold-Containing Silica Aerogels. Gels.

[41] Choi S.M., Kang S.H. (2023). Gold Nanoparticle Superlattice Embedded in Porous Silica and Method for Manufacturing Same. U.S. Patent.

[42] Yin Y., Gao C. (2018). Templated Synthesis of Metal Nanorods in Silica Nanotubes. U.S. Patent.

[43] Ostafin A.E., Nooney R., Maginn E. (2005). Process for Making Mesoporous Silicate Nanoparticle Coatings and Hollow Mesoporous Silica Nano-Shells. U.S. Patent.

[44] Kang S.H. (2022). Metallic Nanoparticle Catalysts Embedded in Porous Oxide Support, Which Show High Catalytic Activity Even at Low Temperatures. U.S. Patent.

[45] Gregg S.J., Sing K.S.W. (1982). Adsorption, Surface Area and Porosity. Berichte Der Bunsenges. Phys. Chem..

[46] Thommes M., Kaneko K., Neimark A., Olivier J.P., Rodriguez-Reinoso F., Rouquerol J., Sing K.S.W. (2015). Physisorption of gases, with special reference to the evaluation of surface area and pore size distribution (IUPAC Technical Report). Pure Appl. Chem..

[47] Shandurkov D., Ignatov P., Spassova I., Gutzov S. (2021). Spectral and Texture Properties of Hydrophobic Aerogel Powders Obtained from Room Temperature Drying. Molecules.

[48] Sing K.S.W., Williams R.T. (2004). Physisorption Hysteresis Loops and the Characterization of Nanoporous Materials. Adsorpt. Sci. Technol..

[49] Villarroel-Rocha J., Barrera D., Blanco A.A.G., Jalil M.E.R., Sapag K. (2013). Importance of the αs-plot Method in the Characterization of Nanoporous Materials. Adsorpt. Sci. Technol..

[50] de Boer J.H., Lippens B.C., Linsen B.G., Broekhoff J.C.P., van den Heuvel A., Osinga T.J. (1966). The T-Curve Of Multimolecular N_2_-Adsorption. J. Colloid Interface Sci..

[51] Bustillo M.A., Fort R., Bustillo M. (1993). Specific surface area and ultramicroporosity in polymorphs of silica. Eur. J. Miner..

[52] Wheatley K. (1959). Measurement of the surface area of tridymite. J. Appl. Chem..

[53] Bohren C.F., Huffman D.R., Clothiaux E.E. (2010). Absorption and Scattering of Light by Small Particles.

[54] Labsphere Spectralon® Diffuse Reflectance Standards. https://www.labsphere.com/product/spectralon-diffuse-reflectance-standards/.

[55] Powdercell http://ccp14.cryst.bbk.ac.uk/ccp/web-mirrors/powdcell/a_v/v_1/powder/e_cell.html.

[56] ImageJ https://imagej.net/ij/.

[57] Neimark A.V., Ravikovitch P.I. (2001). Capillary condensation in MMS and pore structure characterization. Micropor. Mesopor. Mater..

