# Supplementary material for: Porous Silica Gels Doped with Gold Nanoparticles: Preparation, Microstructure, Optical and Textural Properties
Source: Gels. 2025 Jun 13;11(6):454. doi: 10.3390/gels11060454 (PMC12191836; doi:10.3390/gels11060454)
Supplement: Supplementary file 1 [file gels-11-00454-s001.zip › gels-3698288-supplementary.pdf]

X-ray diffraction results of the investigated samples. The phase analysis shows a tetragonal low cristobalite (ICSD 34932) content of 74% and a monoclinic low tridymite (ICSD 176) content of 26%, S1.

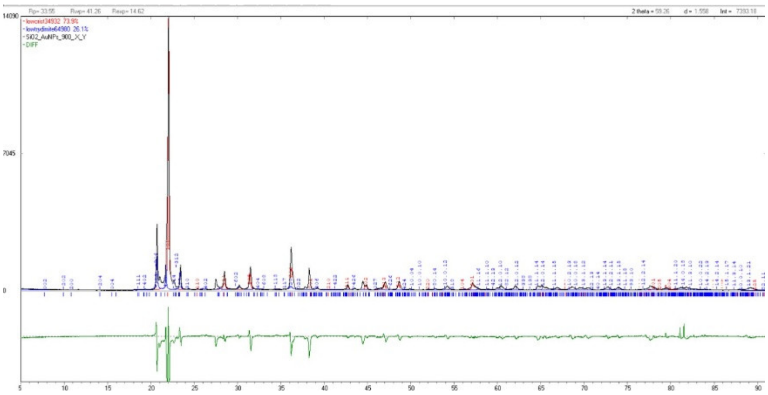

S1 Rietveld analysis of the crystalline sample, T=900 ºC. The phase analysis shows a tetragonal low cristobalite (ICSD 34932) content of 74% and a monoclinic low tridymite (ICSD 176) content of 26%, S1.

The crystallographic data of both phases are from:

Peacor, D. R., High-temperature single-crystal study of the cristobalite inversion, Zeitschrift fuer Kristallographie (144) 1977; ZEKGA 138 (1973) 274-298.

Kato K., Nukuiÿ A., Kristallstruktur des monoklinen Tief-Tridymits, Acta Crystallographica B (24,1968-38,1982); ACBCA 32 (1976) 2486-249.
